# Efficacy of diadynamic currents versus transcutaneous electrical nerve stimulation for transcutaneous tibial nerve stimulation in subjects with overactive bladder syndrome: A double-blind randomized controlled trial

**DOI:** 10.1371/journal.pone.0362118

**Published:** 2026-11-06

**Authors:** Jaya Shanker Tedla, Sneha Latha Lalaji, Ajaya Kumar Midde, Nirmala A. Papalkar, N. Bindhu Priya, Devika Rani Sangadala, Ravi Shankar Reddy, Debjani Mukherjee, Ahlam Mohammed Alamri, Abdullah Suwayyid Alzahrani, Najla Awn Alhadi

**Affiliations:** 1 Department of Medical Rehabilitation Science, College of Applied Medical Sciences, King Khalid University, Abha, Saudi Arabia; 2 Department of Neurorehabilitation, Krishna Institute of Medical Sciences, Secunderabad, Telangana, India; 3 Sr Consultant Urogynecology, Department of Urogynecology, Krishna Institute of Medical Sciences, Secunderabad, Telangana, India; 4 Physiotherapy Department, Armed Forces Hospital Southern Region Rehabilitation Health Services, Khamis Mushait, Saudi Arabia; China Medical University, TAIWAN

## Abstract

**Background:**

In the traditional treatment of overactive bladder (OAB) with transcutaneous posterior tibial nerve stimulation (TTNS), physical therapists currently use only transcutaneous electrical nerve stimulation (TENS). However, the literature has commonly replaced TENS with diadynamic currents for various musculoskeletal pain syndromes. Moreover, no literature describes the use of diadynamic currents in the TTNS procedure. Therefore, our study aimed to determine the efficacy of diadynamic currents (DDC) versus TENS for TTNS in OAB Treatment.

**Methodology:**

A randomized controlled trial was conducted among female patients with OAB aged 18–80 years. Subjects were randomly allocated to control and experimental groups. Participants in the control group received TTNS with TENS, and those in the experimental group underwent TTNS using DDC. Both groups received 8 TTNS sessions, two per week for 4 weeks. We measured pre- and post-intervention improvements using the International Consultation on Incontinence Questionnaire (ICIQ) for OAB and the ICIQ OAB Quality of Life (QOL). The study is registered with the Clinical Trials Registry of India under registration number CTRI/2025/12/099269.

**Results:**

The mean age of the included sample was 59.75 years. Pre- and post-intervention ICIQ-OAB and ICIQ-OAB QOL scores showed significant improvements in disease symptoms and quality of life among patients with OAB in both the control and experimental groups. Comparison of improvements between the two groups on the ICIQ questionnaires showed no significant difference (p-values of 0.87 and 0.95). Correlations between patient-related characteristics and improvements in outcome measures were also not significant.

**Conclusion:**

Eight sessions of TENS and DDC, combined with conventional physical therapy, including Kegel and breathing exercises, can reduce OAB symptoms and improve QOL in subjects with OAB syndrome. Diadynamic current can be used as an adjunct to conventional TENS in TTNS for treating OAB syndrome.

## Introduction

The most acceptable symptoms in the recent definition for the overactive bladder include urge incontinence, frequent urination in daytime and nighttime without any underlying infection or pathology [1]. OAB creates disturbance in activities of daily living and participation, including working, sleep, exercise, and may cause sexual dysfunction [2]. The three standard trio features of OAB are frequent urination, urgency in urination along with incontinence, and nocturia. Nocturia is a primary disturbing clinical feature of OAB, which causes sleep disturbances, poor well-being, and depression in older individuals [3].

Overall, traditional management of OAB includes pharmacologic therapy with beta-3 agonists or antimuscarinic medications, behavioral therapy focused on urge suppression and fluid management, avoidance of bladder irritants (caffeine, alcohol), and incontinence management with coping strategies such as pads, external urine collection systems, absorbent underwear, and conservative physical therapy, including pelvic floor muscle strengthening, biofeedback, TTNS, and magnetotherapy. If conservative measures are not effective, a series of minimally invasive or invasive intervention options is available. Minimally invasive therapies include Botox injections, sacral nerve neuromodulation, TTNS, TNS implants, and indwelling urethral or suprapubic catheters; invasive procedures include urinary diversion or bladder augmentation cystoplasty [4].

TTNS is an application of TENS targeting the posterior branch of the tibial nerve to address OAB clinical features like incontinence, imperative micturition, frequency, and nocturia. TENS with the specified parameters delivers a low-voltage electrical impulse that stimulates the posterior branch of the tibial nerve, a part of the sciatic nerve. This primarily provides motor and sensory innervation to the leg and foot muscles, but also plays an indirect neuromodulatory role in pelvic floor physiology through shared sacral spinal segments S2-S4. [5] Both the sacral and tibial nerves share the same somatic and autonomic innervation, which is also shared by the external urinary sphincter and urinary bladder; hence, it is speculated that neuromodulation of sacral segments may alter reflex urinary pathways via a cross-signaling therapeutic rationale [6].

Dia dynamic currents (DDC) are low-frequency, pulsed, sinusoidal, unidirectional currents obtained by rectifying an alternating current, used to relieve pain and stimulate muscles. Developed by French dentist Pierre Bernard in the 1950s, this therapy treats musculoskeletal disorders by modulating sensory and motor nerves and has been shown to reduce pain. The treatment uses a 50 Hz sinusoidal current with a 10 ms pulse duration and offers five different current types [7].

TENS is a high-frequency bidirectional pulsed current, whereas DDC is unidirectional sinusoidal waves. These currents are commonly compared because of their identical properties, such as depth of penetration, treatment duration, and secure home use [8]. DDCs are most commonly used for musculoskeletal pain, especially low back pain, compared with TENS currents [9,10].

Unlike TENS, DDC is offered in various waveforms and provides a wide range of options based on the patient's needs [11]. DDC waveform shape and current characteristics are expected to enable motor stimulation at lower amplitudes than traditional TENS. Moreover, DDC produces smoother current perception through current modulation and half-wave rectification, thereby reducing skin resistance and making it feel smoother to the subject. DDCs are expected to provide longer therapeutic effects and sustained neuromodulation. At the current frequency of 50–100 Hz, they preferentially activate A-beta and A-delta fibers with lower sensory discomfort, and, due to half-wave rectification, the currents are expected to reach deeper areas [8]. Therefore, in the traditional management of OAB TTNS, physical therapists currently use only TENS currents. However, the literature often replaces TENS with DDC for various musculoskeletal pain syndromes. However, no literature describes the use of DDC in the TTNS procedure.

The current research aims to analyze the effect of DDC compared with TENS on TTNS in OAB treatment and has four objectives. The first objective is to determine the effect of TENS usage in TTNS along with conventional physical therapy (CPT) in reducing symptoms and improving activity and quality of life among subjects with OAB. The second objective is to determine the effect of DDC usage in TTNS along with CPT in reducing symptoms and improving activity and quality of life among subjects with OAB. The third objective is to compare the effect of TENS versus DDC usage in TTNS along with CPT in reducing symptoms and improving activity and quality of life among subjects with OAB. The fourth objective is to determine the correlation between patient-related factors, such as age, height, weight, BMI, duration of disease, and predisposing disorders, with impairments, activity, and quality of life among subjects with OAB. The significance of the research is to demonstrate the effect of DDC usage in TTNS along with CPT in improving impairments, activity, and quality of life among subjects with OAB, to provide an alternative treatment option for subjects with OAB, and to enhance patients’ quality of life by using TTNS for subjects with OAB.

## Methods

This double-blind randomized controlled trial was conducted in accordance with Halinski's ethical principles. The study received ethical committee approval from the Krishna Institute of Medical Sciences (approval number KIMS/EC/BMHR/2024/95-01). The study was registered with the Clinical Trials Registry of India under registration number CTRI/2025/12/099269, and the registration can be found at this URL: https://ctri.nic.in/Clinicaltrials/pmaindet2.php?EncHid=MTQ4NDQ3&Enc=&userName=CTRI/2025/12/099269. We collected the sample using convenience sampling, and we assigned participants to two groups alternately as they were referred to our department. Subjects of the study were people suffering from OAB disorder, and the population included in our study was adult females. Subjects with OAB who had undergone any surgical procedure for the same problem, had metal implants in the tibia and foot, lacked sensation in the foot, or had any other bladder disorder were omitted from the research process.

### Sample size calculation

The sample size was estimated using the clincalc.com sample size calculation [12]. The anticipated mean values used to calculate the sample size were obtained from the literature. In that study, TTNS was the experimental treatment, and ICIQ-OAB was the outcome measure [13]. Based on this study, the group one mean and standard deviation for the main outcome were 8.64 and 3.02, respectively. The anticipated percentage of change in the other group was included. We set the power at 80% (1 – beta), the alpha at 0.05 (the probability of a type I error), the beta at 0.2 (the risk of a type II error), and the group enrollment ratio at 1. The expected sample size was 17 per group, with a 15% dropout rate; we included 20 per group for a total of 40 across the two groups. In this double-blind randomized controlled trial, the experimental and control groups received different treatments.

This research was performed in the Neuro-Rehabilitation Department of Krishna Institute of Medical Sciences, Minister Road Branch, Hyderabad, India. The principal investigator and secondary investigators have more than 10 years of experience in physical therapy and are experts in women's health cases. The research was conducted over 1 year, from 31^st^ January 2025–30^th^ January 2026. The materials required for the study included the Combined Electrotherapy Multicurrent Unit (Enraf Nonius, S.N.10.183, Netherlands), self-adhesive disposable electrodes measuring 5 x 5 cm, a couch, a chair, a table, and office supplies.

The study used the International Consultation on Incontinence Questionnaire for Overactive Bladder (ICIQ-OAB) and the International Consultation on Incontinence Questionnaire for Overactive Bladder Quality of Life (ICIQ-OAB QOL) as outcome measures [14]. ICIQ-OAB is a patient-reported scale with good reliability and validity for assessing the severity and impact of overactive bladder. This scale comprises four items: daytime and nighttime urinary frequency, urgency, and incontinence. Each item is scored for symptom severity and how much this clinical feature bothers the patient. The symptom severity is scored on a maximum of four points; therefore, the total score ranges from 0 to 16, with higher scores indicating greater symptom severity. Each item is scored on a 0–10 scale and is not included in the total severity score; however, it provides deeper insight into patients’ feelings that can inform treatment planning [15].

The International Consultation on Incontinence Questionnaire for Overactive Bladder Quality of Life (ICIQ-OAB QOL) is a patient-reported questionnaire that assesses health-related quality of life in individuals with OAB. The scale comprises 26 items, grouped into domains such as emotions, psychological impact, daily activities, lifestyle limitations, sleep, energy levels, relationships, and general quality of life. The first 25 items are rated on a scale of 1–6, with 1 indicating less impact and 6 indicating a high impact of OAB on their lives. In contrast, the last item is graded on a 0-to-10-point scale that denotes the overall quality of life affected by OAB. The total score for this assessment ranges from 25 to 160, with higher scores indicating greater OAB impact on their lives and lower scores indicating less impact [16].

The study sample was selected through referrals from the Uro-gynecology department. The subjects were informed about the research, and written informed consent was obtained. No coercion was used, and their information was kept confidential throughout. All participants were adult women who could read and understand the English-language questionnaire; the therapist provided any necessary clarifications. The independent investigator, not involved in either treatment or assessment, performed block randomization; the investigator created 4 blocks, each comprising 5 control and 5 experimental subjects. Before subject recruitment, we allocated 10 envelopes per block. We selected subjects in each block using a random chit method and assigned them to a sealed envelope containing a patient sequence number. As patients were selected, we assigned envelopes in sequence, and the group indicated in the concealed envelope was the subject's group. Fig 1 shows the CONSORT flow diagram, including the number of study participants allocated to each analysis group. The control group received TTNS with conventional physical therapy (CPT), and the experimental group received TTNS with DDC in conjunction with CPT.

**Fig 1:**
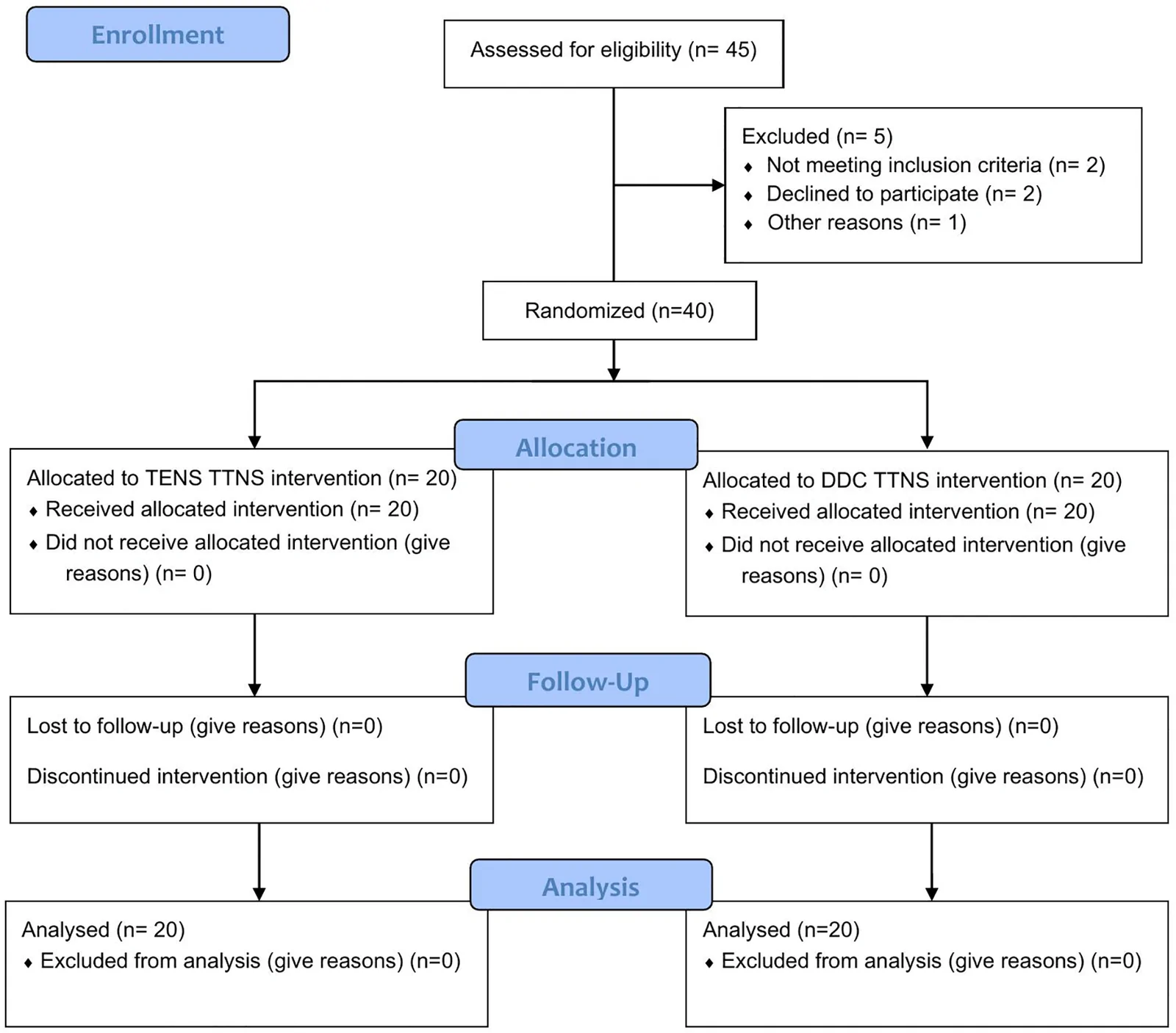
Consort flow diagram showing allotment to analysis details of the study.

Before the procedure, subjects received detailed information about the treatment. Once the subject was in a comfortable position, we gathered the materials required for the stimulation procedure, including the machine, electrodes, sanitizer, and cotton. We performed the TTNS procedure while the subject lay down, and we tested the stimulation site for touch and temperature sensation using a brush and test tubes. Subjects were instructed that they would experience a mild, non-painful tingling sensation from the electrical current and should inform the therapist if they experienced any abnormal sensations, such as heat, burning, pain, or spasm. The stimulation area was cleaned with sanitizer and a cotton swab. Once the area was dry, the self-adhesive electrodes were secured to the leg. The anode was placed on the medial arch of the foot, and the cathode was placed 10 cm above the medial malleolus, superiorly and posteriorly on the same leg. (Fig 2) [17]. The left or right foot was selected according to the subject's preference and the therapist's convenience.

**Fig 2:**
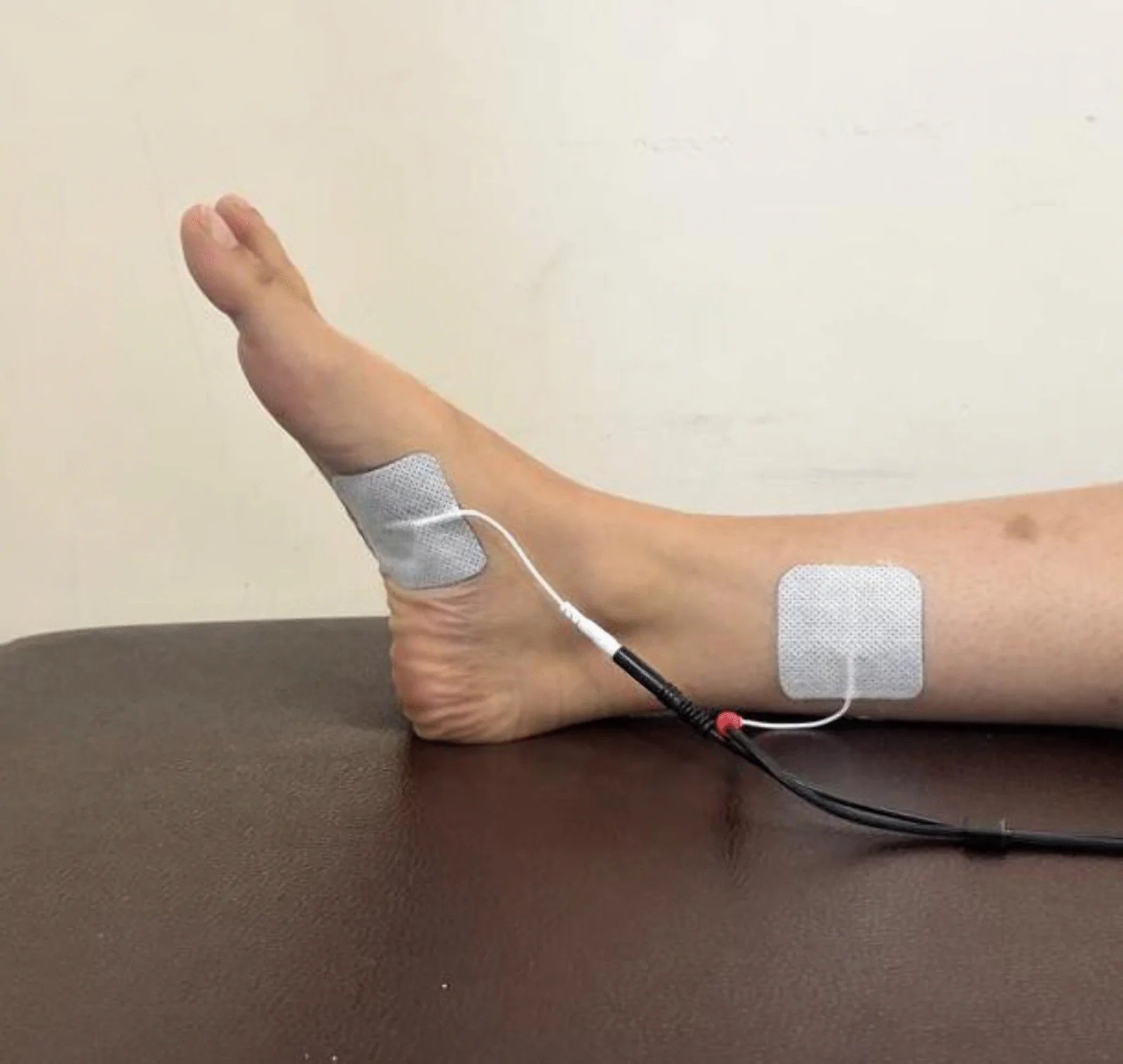
Demonstrating the placement of electrodes for transcutaneous tibial nerve stimulation.

The control group received TTNS with TENS in conjunction with the CPT. TENS is continuous stimulation, at a pulse width of 200 microseconds and a frequency of 10 Hz. We increased the amplitude to a level that produced sensory stimulation in the same foot at a tolerable intensity for the patient; a commonly used range was 30–70 mA [18]. Typically, we increased the intensity gradually and aimed to reach a level that elicited a motor response of great-toe extension. The therapy consists of 30 minutes of TENS and 30 minutes of CPT, delivered twice per week for 4 weeks, for a total of 8 sessions.

The intervention in the experimental group was TTNS with DDC, administered in conjunction with the CPT. DDC stimulation was performed using Diphase Fixe/Diphase (DF) at 100 Hz in surge mode, with a pulse width of 200 microseconds to 1 second and an intensity typically adjusted to the subject's comfort [7,10]. We commonly increased the intensity gradually to elicit a motor response, then adjusted it to the subject’s comfort. For both TENS and DDC treatments, the same electrode placement was used. The therapy consists of DDC and CPT, delivered as a one-hour session twice per week for four weeks, for a total of 8 sittings.

Conventional physical therapy interventions include Kegel exercises and breathing exercises. The subjects will perform Kegel exercises for 15 minutes and breathing exercises for another 15 minutes, twice per week for four weeks, for a total of eight sessions in front of the therapist. The home program included Kegel exercises: 10 repetitions with a 5-second hold, performed three times daily, along with deep-breathing exercises.

While performing Kegel exercises, the patient can assume a supine, sitting, or crook-lying position and ensure the bladder is empty. Contract the pelvic floor muscles as if holding urine. Hold for 5 sec, then relax for 10 sec and repeat the contraction. Do 10 sets of contraction and relaxation for three sessions in a day [19,20]. Breathing is prescribed along with Kegels for practical pelvic floor training. In this position, the patient lies supine with hips and knees flexed, feet resting flat on the surface, hands by the sides of the body, spine neutrally aligned and slightly lengthened, and neck and shoulders relaxed. Slightly widen the rib cage. Relax the abdominal and pelvic floor muscles. Inhale through the nose, concentrating on widening the rib cage. The abdomen should not rise while inhaling. After complete inhalation, hold for 15 sec, then slowly exhale through the mouth. Repeat this for 10 repetitions, three times a day [19].

In both groups, therapy lasted 60 minutes per session, for a total of 8 sessions over 4 weeks. Both groups also had the same home program of Kegels and breathing exercises. We collected outcome measures pre- and post-intervention over 4 weeks.

### Data analysis

Data Analysis was performed using the Statistical Package for the Social Sciences (version 27; IBM, International Business Machines Corporation, Armonk, New York, USA) and RStudio (version 2025; Posit, PBC, Boston, USA). Descriptive statistics (mean, standard deviation, minimum, and maximum) for parameters such as age, height, weight, body mass index, duration of OAB, duration of diabetes, and duration of hypertension, along with outcome measures, were obtained using univariate analysis. We analyzed pre-post differences using the Wilcoxon signed-rank test and between-group differences using the Mann-Whitney U test. P-values were kept at 0.05 to detect significant differences at the 95% confidence interval. Linear mixed model analysis was conducted using the “lme” package in R.

## Results

In this double-blind randomized controlled trial, we compared two active group treatments using TENS and DDC currents in subjects with OAB, using the ICIQ-OAB and QOL questionnaires. Table 1 compares the baseline physical characteristics, such as weight, height, and body mass index, of the two groups, showing good homogeneity. The participants were middle-aged to older females with or without comorbidities such as hypertension and diabetes, and the duration of OAB was evenly distributed between the groups. The history of diabetes was positive for 28 people and negative for 12 people. The history of hypertension was positive in 25 people and negative in 15, for a total sample of 40. Baseline assessment of overactive bladder symptoms using the ICIQ-OAB and QOL questionnaires indicated somewhat higher scores in the experimental group. Post-interventional assessment showed marked improvement in OAB symptoms. The QOL scores improved from baseline to post-interventional assessment and increased substantially in both groups. This information indicates a sustainable improvement in the subject's well-being, regardless of group assignment.

**Table 1:** Demographic characteristics, baseline, and post-interventional scores of ICIQ-OAB and ICIQ-QOL in experimental and control groups.

Patient parameters	Total (M ± SD)	Control (M ± SD)	Experimental (M ± SD)
Height (Meters)	1.64 ± 0.09	1.65 ± 0.10	1.63 ± 0.08
Weight (KG)	74.28 ± 10.56	74.35 ± 11.50	74.20 ± 9.83
BMI	27.79 ± 4.13	27.50 ± 4.25	28.09 ± 4.09
Duration of OAB (Months)	17.30 ± 11.92	17.05 ± 13.52	17.55 ± 10.41
Duration of diabetes (Years)	7.68 ± 6.76	6.50 ± 7.09	8.85 ± 6.38
Duration of Hypertension (Years)	5.93 ± 5.82	5.05 ± 6.22	6.80 ± 5.40
Age (years)	59.75 ± 14.90	58.30 ± 17.57	61.20 ± 11.93
Pre ICIQ-OAB (16)	11.98 ± 2.68	11.10 ± 2.95	12.85 ± 2.11
Post ICIQ-OAB (16)	3.93 ± 3.13	3.15 ± 2.46	4.70 ± 3.59
Pre ICIQ-OAB QOL (160)	113.48 ± 28.09	104.65 ± 33.44	122.30 ± 18.37
Post ICIQ-OAB QOL (160)	55.20 ± 28.72	46.05 ± 15.50	64.35 ± 35.73
ICIQ – OAB Pre-Post	8.05 ± 4.03	7.95 ± 3.73	8.15 ± 4.40
ICIQ – OAB QOL Pre-Post	58.28 ± 32.33	58.60 ± 3.32	57.95 ± 32.16

Note: M: mean, KG: kilograms, SD: standard deviation, OAB: overactive bladder, ICIQ-OAB: International Consultation on Incontinence Questionnaire – Overactive Bladder, QOL: quality of life.

Table 2 presents the pre- and post-interventional comparative outcomes for both the control and experimental groups, focusing on participants’ symptoms and well-being with OAB syndrome. The results are expressed as the mean values with standard deviations along with corresponding p-values. ICIQ-OAB and ICIQ-OAB QOL scores improved significantly in both groups, indicating that the intervention period was associated with meaningful clinical improvement. This improvement was statistically significant (p < 0.001), providing strong evidence against chance findings.

**Table 2:** Comparison of Pre and Post Intervention ICIQ-OAB and ICIQ OAB QOL Scores Between Control and Experimental Groups.

Group	Pair	ICIQ Outcome	Mean ± SD	p value
Control	Pair 1	Pre ICIQ-OAB (16)	11.1 ± 2.95	<0.001
		Post ICIQ-OAB (16)	3.15 ± 2.45	
	Pair 2	Pre ICIQ-OAB QOL (160)	104.65 ± 33.44	<0.001
		Post ICIQ-OAB QOL (160)	46.05 ± 15.50	
Experimental	Pair 1	Pre ICIQ-OAB (16)	12.85 ± 2.10	<0.001
		Post ICIQ-OAB (16)	4.7 ± 3.58	
	Pair 2	Pre ICIQ-OAB QOL (160)	122.3 ± 18.36	<0.001
		Post ICIQ-OAB QOL (160)	64.35 ± 35.73	

Note: SD: standard deviation, OAB: overactive bladder, ICIQ-OAB: International Consultation on Incontinence Questionnaire – Overactive Bladder, QOL: quality of life, p-value represents the level of significance between the pre- and post-outcome difference, Pair: pre- and post-values of one outcome that is compared.

Table 3 presents the pre-post mean differences in ICIQ-OAB and ICIQ-OAB QOL scores between the control and experimental groups. However, there is no statistically significant difference in improvement results between the experimental and control groups. The p-values are 0.878 for the ICIQ-OAB scores and 0.950 for the ICIQ-OAB QOL scores.

**Table 3:** Comparison between the groups in ICIQ-OAB and ICIQ-OAB QOL scores.

Outcome measure	Group	Mean ± SD	p value
ICIQ – OAB Pre-Post	Control	7.95 ± 3.73	0.878
	Experimental	8.15 ± 4.40	
ICIQ – OAB QOL Pre-Post	Control	58.60 ± 33.31	0.950
	Experimental	57.95 ± 32.16	

Note: SD: standard deviation, OAB: overactive bladder, ICIQ-OAB: International Consultation on Incontinence Questionnaire – Overactive Bladder, QOL: quality of life.

The correlation table indicates that most demographic variables, such as height and weight, and comorbidities such as diabetes and hypertension status and duration, do not correlate with improvement in ICIQ-OAB and ICIQ-OAB QOL scores after intervention in subjects with OAB. However, the duration of OAB disease has a mildly significant correlation with improvements in quality of life, and a history of diabetes has a mildly significant correlation with symptoms of OAB. The details of these R and p values are shown in Table 4.

**Table 4:** Correlation of Demographic and Clinical Parameters with Post-Intervention ICIQ OAB Symptoms and Quality of Life Outcomes.

Correlation Parameters	r or p value	Post ICIQ-OAB (16)	Post ICIQ-OAB QOL (160)	ICIQ- OAB Pre-Post	ICIQ-OAB QOL Pre-Post
Height (Meters)	r value	0.228	0.195	-0.176	-0.093
	p value	0.158	0.228	0.277	0.567
Weight (Kilogram)	r value	0.023	0.065	-0.152	0.01
	p value	0.887	0.692	0.351	0.953
Body mass index	r value	-0.145	-0.085	-0.028	0.077
	p value	0.373	0.602	0.866	0.638
Duration of OAB (Months)	r value	-0.169	-0.134	0.265	.444**
	p value	0.297	0.409	0.099	0.004
History of Diabetes	r value	0.069	-0.135	-.365*	-0.122
	p value	0.673	0.405	0.021	0.454
Duration of Diabetes (Years)	r value	-0.01	0.18	0.294	0.066
	p value	0.953	0.267	0.066	0.687
History of Hypertension	r value	-0.065	0.002	-0.088	-0.11
	p value	0.692	0.991	0.591	0.498
Duration of Hypertension (Years)	r value	0.225	0.078	0.012	0.038
	p value	0.163	0.632	0.940	0.816
Age	r value	-0.188	0.022	0.302	0.029
	p value	0.246	0.891	0.058	0.860

Note: * Correlation is significant at the 0.05 level (2-tailed), ** Correlation is significant at the 0.01 level (2-tailed), OAB: overactive bladder, ICIQ-OAB: International Consultation on Incontinence Questionnaire – Overactive Bladder, QOL: quality of life.

To understand which treatment has more beneficial effects in both ICIQ OAB and ICIQ OAB QOL outcomes. Changes in questionnaire scores were analysed using a linear mixed-effects model with fixed effects for Treatment Group, Pre-Post Time, and Outcome (QOL160, ICIQ-OAB16), and their interactions, and a random intercept for subject. The model showed a significant effect of Time (F(1,120) = 153.58, *p* < 0.001), indicating substantial improvement from pre- to post-intervention across both treatment groups. The Time × Group interaction was not significant (F(1,120) = 0.02, *p* = 0.88), indicating no differential treatment effect between groups. Improvement differed between outcome measures (Time × Outcome: F(1,120) = 87.21, *p* < 0.001), reflecting differences in sensitivity to change between QOL160 and ICIQ-OAB16; however, the three-way interaction (Time × Group × Outcome) was not significant (F(1,120) = 0.01, *p* = 0.91), indicating that relative treatment effects were consistent across outcomes. Fig 3 shows the linear mixed model details, including the effects of time, outcome, and group interactions.

**Fig 3:**
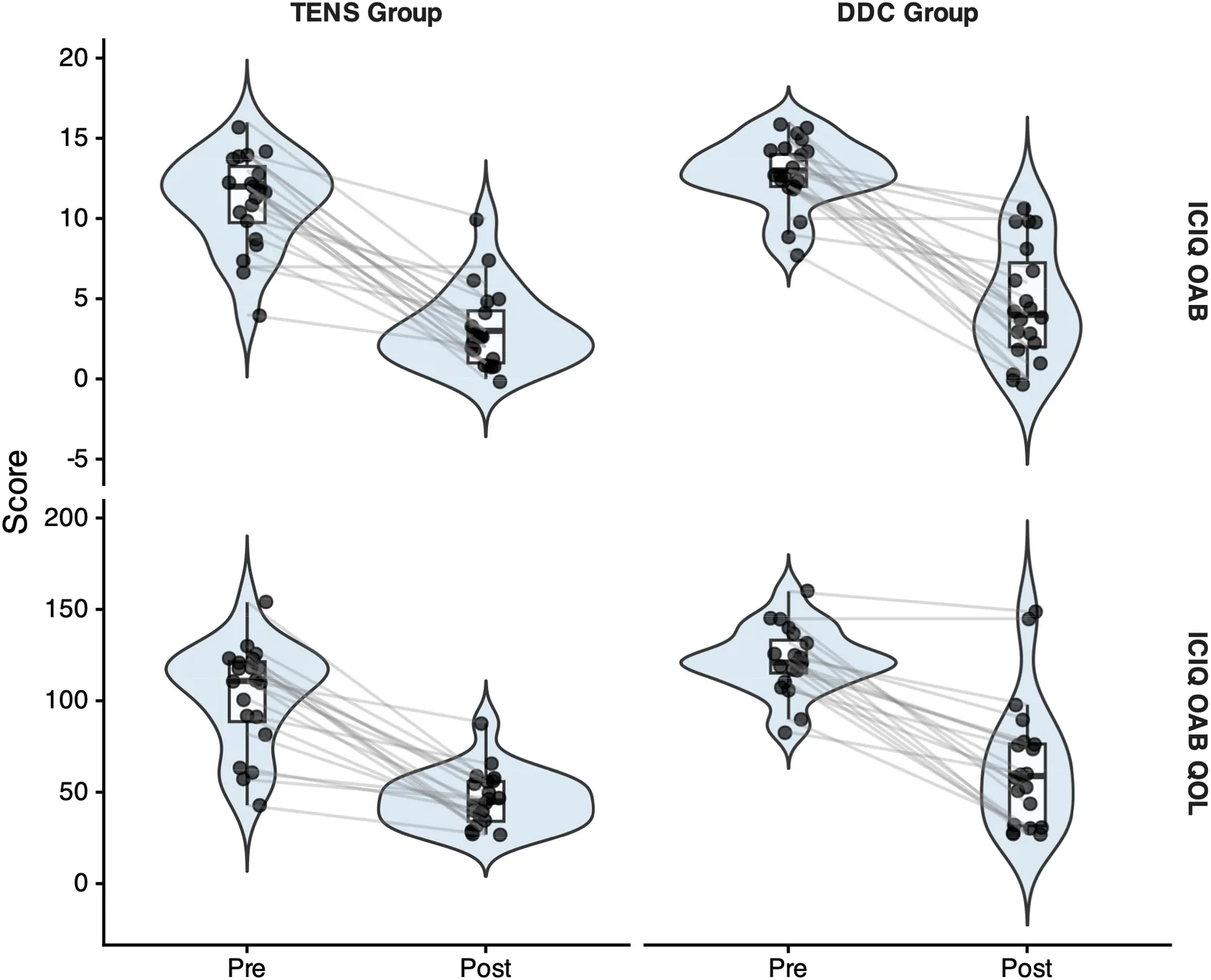
Graph showing the impact of time, outcome measure and treatment group linear mixed model analysis results.

## Discussion

In this research, we intended to investigate the suitability of DDC for TTNS as an alternative to TENS for treating patients with OAB. After statistical analysis, the p-values for ICIQ OAB and ICIQ OAB QOL pre- and post-TTNS in the experimental group managed with DDC and the control group managed with TENS show a significant difference, indicating that both treatment options are effective in improving OAB. The improvement comparison between DDC and TENS for TTNS shows no statistically significant difference, suggesting that DDC can be used as an alternative to TENS for TTNS in patients with OAB. In this study, DDC has been systematically compared with TENS for TTNS for the first time. Thus, the clinically significant improvement in this study demonstrates the increase in treatment options for noninvasive neuromodulation to treat OAB.

Most of the time, females are more affected by OAB than males, and that is the reason why, in our sample, we have included only female subjects [20,21]. The mean age of the subjects was 59 years or older, and we intended to take a sample from 18 to 80 years old, but most were near 60 years old, and this observation has also been noted in previous studies [22]. Overall, body weight and body mass index also played a crucial role in OAB [23]. In our research, the average BMI was more than 27, indicating most of the sample was overweight or obese. This supports the hypothesis that overweight or obese individuals are prone to OAB. Comorbidities, such as diabetes and hypertension, were also strongly associated with disease incidence [24,25], and we observed that the majority of our sample had either diabetes or hypertension. The average values of ICIQ OAB and ICIQ OAB QOL in our research are also like those represented in the literature for subjects with OAB [14,26,27].

TTNS with TENS has been proven to significantly improve OAB through neuromodulation of bladder function by stimulating the posterior tibial nerve, which inhibits detrusor overactivity and reduces symptoms such as urgency and frequency by stimulating spinal pathways via the L5-S3 nerve root segments. Many randomized controlled trials, systematic reviews, and meta-analyses have also proven the effect of TENS in TTNS for subjects with OAB [18,28,29].

Both DDC and TENS are noninvasive electrotherapy modalities that deliver current through surface electrodes applied to the skin. Both modalities are known for their analgesic effects, or pain modulation, which mainly act through the pain gate mechanism and the activation of pain-inhibiting fibers [10]. In a study performed by Demidas et al., 44 patients were divided equally into two groups. One group received DDC and the other TENS to assess touch and pressure sensations. Touch was measured using an esthesiometer, whereas pressure was measured with an algometer. DDC, which is shorter in duration, is a suitable adjunct to TENS, as both produced similar analgesic effects [8]. Similarly, TENS and DDC were compared in many conditions, such as patellofemoral pain syndrome, low back pain, and temporomandibular joint pain, etc., and produced similar effects [7,30,31].

DDC delivers low-frequency sinusoidal currents in various patterns, such as monophasic fixed, diphase fixed, short periods, long periods, etc., and these dynamic currents provide a broader spectrum of afferent stimulation than TENS. This generates a continuous, rhythmic, modulated stimulus that produces a more substantial, sustained afferent nerve-activating effect than the pulsed, biphasic waveform of conventional TENS. Because DDC is direct current, it may provide continuous stimulation, and a variety of afferent stimuli may have a longer-lasting effect than traditional pulsed TENS. Because DDCs vary widely, stimulation can be customized to provide better sensory feedback for the individual. Moreover, DDC treatment is shorter than conventional TENS in subjects with OAB. For these reasons, DDC has produced effects similar to TENS in controlling OAB [7–9,30,31].. DDC-type continuous stimulation would have aided segmental and suprasegmental inhibition of detrusor overactivity by prolonging activation of A-β and A-δ afferent fibers. This is how bladder reflex modulation is hypothesized to be achieved with DDC-like TENS currents.

### Limitations and future suggestions of the study

We understand that the study has a small sample size, but future studies should consider our limitations and increase the number of subjects in multicentric models by using larger, more diverse cohorts to further validate the independent use of DDC as a treatment measure. The outcomes used in this study are **questionnaire-based scales;** although validated and clinically relevant, these are subjective. The use of an objective quantitative outcome measure, such as urodynamic studies, is preferable for enhancing the efficacy of the results. The current study was double-blind for the assessor and the participant; however, the therapist who was delivering the treatment was not blinded to the groups. Hence, making the study triple-blinded, with the therapist blinded as well, may enhance the study’s results. This study is a single-center study, which may limit the external validity and generalizability of the findings to broader clinical settings. This study does not contain a pure control or sham TTNS group. This limits the ability to detect and differentiate treatment responses from natural improvements and the placebo effect of TTNS in OAB.

Including objective outcome measures, such as urodynamic studies, and conducting a triple-blinded, multicenter, randomized controlled trial with pure control and placebo groups may strengthen the evidence for the use of DDC in TTNS as an alternative to TENS. A triple-blinded method is recommended for subsequent trials in which participants, investigators, and assessors remain blinded, thereby enhancing the objectivity of outcome interpretations. Incorporating a comparator study that compares DDC with conventional physiotherapy as a pure control will demonstrate the incremental benefits of DDC over standard physiotherapy alone in OAB. Inclusion of a sham treatment group for TTNS would help better detect the true TTNS effect from placebo responses. A multicenter study design ensures a large, diverse cohort, and its results will further enhance the validity of DDC use. Alternative electrotherapeutic approaches, such as electro-galvanic baths, faradic currents, and other neuromodulation modalities, may also have effects similar to those in our study, providing additional treatment options and increasing the suitability of TTNS for many individuals affected by OAB.

## Conclusion

Diadynamic currents, or TENS currents, when combined with conventional physical therapy, including Kegel and breathing exercises, showed improvements as determined by statistical analysis. This indicates that eight sessions of TENS or DDC combined with CPT can reduce the OAB symptoms and improve the QOL in subjects with OAB syndrome. Diadynamic current can be used as an adjunct to conventional TENS in TTNS to manage OAB syndrome.

## Supporting information

S1 File Consort for interventional studies 3-5. (PDF)

S2 File PLOS Human Participants Research Checklist 2025. (PDF)

S3 File TTNS praposal. (DOCX)
