# Supplementary material for: Efficacy of diadynamic currents versus transcutaneous electrical nerve stimulation for transcutaneous tibial nerve stimulation in subjects with overactive bladder syndrome: A double-blind randomized controlled trial
Source: PLoS One. 2026 Nov 6;21(11):e0362118. doi: 10.1371/journal.pone.0362118 (PMC13742903; doi:10.1371/journal.pone.0362118)
Supplement: Supplementary file 3 [file pone.0362118.s003.docx]

### Efficacy of Diadynamic Currents versus Transcutaneous Electrical Nerve Stimulation for Transcutaneous Posterior Tibial Nerve Stimulation in subjects with Overactive Bladder syndrome. A double-blinded randomised controlled trial.

**Abstract**

Background: In the traditional management of overactive bladder (OAB) with transcutaneous posterior tibial nerve stimulation (TTNS), physical therapists currently use only transcutaneous electrical nerve stimulation (TENS) currents. However, TENS is commonly replaced in the literature by diadynamic currents for various musculoskeletal pain syndromes. Moreover, there is no literature on the use of diadynamic currents in the TTNS procedure. Hence, the aim of our study was to determine the efficacy of diadynamic currents (DDC) versus TENS for TTNS in OAB Treatment.

Methodology: A randomised controlled trial will be conducted in female patients with OAB aged 18 to 80 years. Participants will be randomly assigned to control and experimental groups. Participants in the controlled group will receive TTNS with TENS, and participants in the experimental group will receive TTNS with DDC. Both groups will receive 8 sessions of TTNS twice a week for 4 weeks. Pre and post-intervention improvement will be measured using the International Consultation on Incontinence Questionnaire (ICIQ) for OAB and ICIQ OAB Quality of Life (QOL).

Keywords: overactive bladder syndrome, transcutaneous posterior tibial nerve stimulation, transcutaneous electrical nerve stimulation, diadynamic currents, quality of life, incontinence.

**Introduction**

The most acceptable recent definition for the overactive bladder is ‘‘overactive bladder syndrome is characterized by urinary urgency, with or without urgency urinary incontinence, usually with increased daytime frequency and nocturia, if there is no proven infection or other obvious pathology’’.(1) OAB create disturbance in daily activities and social activities such as working, exercise, sleep, and may cause sexual dysfunction. (2) The three common trio features of OAB are frequent urination, urgency in urination with or without incontinence, and nocturia. Nocturia is one of the most common and most disturbing symptoms of OAB, which causes sleep disturbances, poor quality of life, and depression in the elderly population. (3)

Over all the traditional management of OAB includes pharmacological management with beta three agonists or antimuscarinic medications, behavioural therapy using suppression of urge, fluid management, avoiding bladder irritants (caffeine, alcohol), management of incontinence by coping strategies including pads, external urine collection system, absorbent underwear and conservative physical therapy management including pelvic floor muscle training, biofeedback, trans cutaneous tibial nerve stimulation, and electromagnetic therapy. If conservatively the problem is not manageable, then there are series of intervention choices which are minimal invasive or invasive therapy options. Minimal invasive therapies are botulinum toxin injection of the bladder, sacral neuro modulation, percutaneous tibial nerve stimulation, implantable tibial nerve stimulation, indwelling urethral or supra pubic catheter, and invasive procedures are urinary diversion or bladder augmentation cystoplasty. (4)

TTNS is an application of TENS (transcutaneous electrical nerve stimulation) targeting the posterior tibial nerve to address symptoms of overactive bladder (OAB), including urinary incontinence, urgency, frequency, and nocturia. TENS with the specific parameters (i.e. frequency 10-20 Hz pulse width 200-300 microseconds and intensity to motor thresholds for a duration of 30 minutes) will deliver low voltage electrical impulse that stimulate the posterior tibial nerve which is a branch of sciatic nerve, this primarily provide motor and sensory innervation to leg and foot muscles but plays an indirect neuromodulation role in pelvic floor physiology through shared sacral spinal segments S2-S4.(5) As the tibial nerve shares segmental innervation with the autonomic and somatic nerves of sacral segments, which also innervate the bladder and external sphincter, it has been speculated that neuromodulation of sacral segments may alter voiding reflex pathways through cross-signaling mechanism.(6)

Dia dynamic currents (DDC) are low-frequency, pulsed, sinusoidal, unidirectional currents obtained by rectifying an alternating current, used to relieve pain and stimulate muscles. Developed by French dentist Pierre Bernard in the 1950s, this therapy is used to treat musculoskeletal disorders by influencing sensory and motor nerves and has been shown to reduce pain. The treatment uses a 50 Hz sinusoidal current with a 10 ms pulse duration and offers five different current types. (7) TENS is a high-frequency, bidirectional, pulsed current, whereas DDC is unidirectional, sinusoidal waves; these currents are commonly compared because of their similar properties, such as depth of penetration, treatment duration, and secure home use. (8) DDC are most commonly used in musculoskeletal pain, especially in low back pain, in comparison with TENS currents. (9, 10)

DDC is offered in various versatile waveforms, unlike TENS, and offers a wide range of selection types based on the patient's needs. (11) DDC waveform shape and current characteristics are expected to do motor stimulation at lower amplitudes compared to traditional TENS. Moreover, DDC produces smoother current perception due to current modulation and half-wave rectification, which reduces skin resistance and thus feels smoother to the subject. DDC are expected to produce longer therapeutic effects and sustained neuro-modulation. At the current frequency of 50-100 Hz, they preferentially activate A-beta and A-delta fibres with lower sensory discomfort, and, due to half-wave rectification, the currents are expected to reach deeper areas. (12)Hence, in the traditional management of OAB TTNS, currently, the physical therapists are using only TENS currents. However, TENS is commonly replaced in the literature by DDC for various musculoskeletal pain syndromes. However, there is no literature available on the use of DDC in the TTNS procedure.

The Aim of the study was to analyse the efficacy of DDC versus TENS for TTNS in OAB Treatment, and the study has four objectives. First one is to find out the effect of TENS usage in TTNS along with conventional physical therapy (CPT) in improving impairments, activity and quality of life among subjects with OAB, second one is to find out the effect of DDC usage in TTNS along with CPT in improving impairments, activity and quality of life among subjects with OAB, Third one is to compare the effect of TENS versus DDC usage in TTNS along with CPT in improving impairments, activity and quality of life among subjects with OAB, and fourth one is to find out the correlation between patient-related factors like age, height, weight, BMI, duration of disease, predisposing disorders with impairments, activity and quality of life among subjects with OAB. The significance of the study was to demonstrate the efficacy of DDC usage in TTNS along with CPT in improving impairments, activity, and quality of life among subjects with OAB, providing the alternative treatment availability for subjects with OAB, and to enhance the patients' quality of life by using electrical stimulation as an adjunct treatment for subjects with OAB.

**Methods**

This quasi-randomised controlled trial is conducted in accordance with Halinski's ethical principles. The study obtained ethical committee approval from the Krishna Institute of Medical Sciences, with approval number KIMS/EC/BMHR/2024/95-01. The sample was collected using convenience sampling, and participants were distributed into two groups alternately as they were referred to our department. Subjects of the study were people suffering from overactive bladder disorder (OABD), and the population included in our study was adult females. The subject of OABD who had undergone any surgical procedure for the same problem, had metal implants in the tibia and foot, lacked sensation in the foot, or had any other bladder disorder were excluded from the study process.

The sample size for the study was calculated by using the clincalc.com sample size calculation. (13) In this quasi-experimental study design, the experimental and control groups receive different treatments. The anticipated mean values used to calculate sample size were taken from the literature, where TTNS was the experimental treatment and ICIQ-OAB the outcome measure. (14) Based on this study, we set the power at 80%, the probability of type 1 error (alpha) at 0.05, the probability of type 2 error (beta) at 0.2, and the enrollment ratio between the groups at 1. The expected sample size was 27 per group, including a 10% dropout rate; we expected a sample size of 30 per group, and across the two groups, we recruited 60 members in total.

The study will be conducted in the Rehabilitation Department of Krishna Institute of Medical Sciences, Minister Road Branch, Hyderabad, India. The principal investigator and secondary investigators have more than 10 years of experience in physical therapy and are experts in women's health cases. The study was conducted for 1 year, from December 2024 to November 2025. The materials required for the study included the Combined Electrotherapy Unit from Enraf Nonius self-adhesive disposable electrodes of 5X5 size, a couch, a chair, a table, and some office materials.

The outcome measures used in the study willbe the International Consultation on Incontinence Questionnaire for Overactive Bladder (ICIQ-OAB) and the International Consultation on Incontinence Questionnaire for Overactive Bladder Quality of Life (ICIQ-OAB QOL). (15) The International Consultation on Incontinence Questionnaire for Overactive Bladder (ICIQ-OAB) is a valid and reliable patient-reported questionnaire used to assess the severity and impact of overactive bladder. This scale has four items: daytime urinary frequency, nighttime urinary frequency, urgency, and urinary incontinence. Each item is scored for symptom severity and the extent to which this clinical feature bothers the patient. The symptom severity is scored on a maximum of four points; hence, four items together will make a minimum to maximum score of zero to 16, and the higher the score, the more severe the symptom severity. Each item is scored on a 0-10 scale and is not included in the total severity score; however, it provides deeper insight into patients' feelings, which can be used to plan treatment. (16)

The International Consultation on Incontinence Questionnaire for Overactive Bladder Quality of Life (ICIQ-OAB QOL) is a patient-reported questionnaire used to measure health-related quality of life among subjects with overactive bladder syndrome. The scale comprises 26 items, grouped into domains such as emotions, psychological impact, daily activities, lifestyle limitations, sleep, energy levels, relationships, and general quality of life. The 1st 25 items are measured on a scale of one to six. Where one indicated less impact, six indicated a high impact of the OAB on their life. In contrast, the last item is measured on a 0-to-10-point scale that denotes the overall quality of life affected by OAB. The total score of this scale ranges from 25 to 160, with higher scores indicating greater OAB impact on their lives and lower scores indicating less impact. (17)

The study sample will be selected from referrals to our department from the Uro-gynaecology department. The subjects will be informed about the study, and written informed consent will be obtained. No force would be imposed on the subjects, and their information will be kept confidential throughout. All participants would be adult women who could read and understand the questionnaire in English; the therapist provided any clarifications needed. The subjects will be divided into a control group using TTNS (Transcutaneous Electrical Nerve Stimulation) in conjunction with conventional physical therapy (CPT) and an experimental group using TTNS (Dia Dynamic currents) in conjunction with CPT.

The subjects will be informed in detail about the treatment procedure before undergoing it. Once the subject is in a comfortable position, we will collect the materials required for the stimulation procedure, such as the machine, electrodes, sanitizer, and cotton. The TTNS procedure was performed in the supine position, and the area of stimulation was tested for touch and temperature sensations using a brush and test tubes. The subjects will be instructed that they would feel a mild tingling sensation from the electrical current, which is not painful, and that they should inform the therapist if they experienced any abnormal sensations, such as heat, burning, pain, or spasm. The stimulation area was cleaned with sanitizer and cotton. Once the area was dry, the self-adhesive electrodes will be secured to the leg. The anode will be kept on the medial arch of the foot, and the cathode will be placed 10 cm above the medial malleolus superiorly and posteriorly on the same foot. (18) The left or right foot is chosen based on the subject's preference and the therapist's convenience.

Intervention in the control group was TTNS with Transcutaneous Electrical Nerve Stimulation (TENS) in conjunction with the CPT. Transcutaneous electrical nerve stimulation is continuous stimulation, at a pulse width of 200 microseconds and a frequency of 10Hz. The amplitude will be set to produce a sensory stimulus in the ipsilateral foot, at an intensity tolerable to the patient; commonly used range was 30 to 70 mA. (19) Usually, we will increase the intensity gradually and will try to reach a level at which we elicited a motor response of great toe extension. The therapy consists of 30 minutes of TENS and 30 minutes of CPT, delivered twice a week for 4 weeks, for a total of 8 sessions.

Intervention in the experimental group was TTNS with DD currents in conjunction with the CPT. DD current stimulation will be performed at 10 Hz, with a pulse width of 200microseconds and an intensity usually between 30 and 70 milliamperes, based on the subject's comfort. Commonly, we increased the intensity gradually and tried to reach a level at which we elicited a motor response of great toe extension. The therapy consists of 30 minutes of DD currents and 30 minutes of CPT, two sessions per week for four weeks, for a total of 8 sessions.

Intervention in the conventional physical therapy includes Kegel exercises and breathing exercises. The subjects will perform Kegel exercises for 15 minutes and breathing exercises for another 15 minutes, two sessions per week for four weeks, for a total of eight sessions in front of the therapist. The home program included Kegel exercises: 10 repetitions with a 5-second hold, three times a day, along with deep-breathing exercises.

While performing the Kegel exercises, the patient will assume either the supine, sitting, or crook-lying position and ensure that the bladder is empty. Contract the pelvic floor muscles as if holding urine. Each hold will be for 5 seconds, then let go, relax for 10 seconds, and repeat the contraction. Do 10 sets of contraction and relaxation for 3 sessions in a day. (20, 21) Breathing is prescribed along with Kegels for effective pelvic floor training. In this the patient is asked to lie down supine with hips and knees flexed feet resting flat on surface hands by the side of the body spine neutrally aligned and lengthened slightly, neck and shoulders relaxed, the rib cage should be slightly widened abdominal and pelvic floor muscles should be relaxed inhalation through nose concentrate on widening of rib cage on inhalation the abdomen should not raise while inhaling after complete inhalation hold for 15 sec then slowly exhale through mouth repeat this for 10 repetitions three times a day. (22)In both groups, the therapy lasted 60 minutes by the therapist for a total of 8 sessions, over a period of four weeks. Both groups also had the same home program of Kegels and breathing exercises. The outcome measures were collected pre- and post-intervention for 4 weeks.

Data Analysis was conducted using the Statistical Package for the Social Sciences (version 27; IBM, International Business Machines Corporation, Armonk, New York, USA) and RStudio software (version 2025; Posit, PBC, Boston, USA). The descriptive statistics, like mean, standard deviation, minimum, and maximum for parameters like age, height, weight, body mass index, duration of OAB, duration of diabetes, and duration of hypertension, along with outcome measures, were obtained by univariate analysis in descriptive statistics. The pre-post differences were analysed using the Wilcoxon signed-rank test, and the group differences were analysed using the Mann-Whitney U test. Level of significance (p-value) kept at 0.05 for significant differences at a 95% confidence interval. Linear mixed model analysis was performed using the “lme” package in R.

**References**

[1] M.J. Drake, Do we need a new definition of the overactive bladder syndrome? ICI‐RS 2013, Neurourology and urodynamics 33(5) (2014) 622-624.

[2] B.T. Haylen, D. De Ridder, R.M. Freeman, S.E. Swift, B. Berghmans, J. Lee, A. Monga, E. Petri, D.E. Rizk, P.K. Sand, An International Urogynecological Association (IUGA)/International Continence Society (ICS) joint report on the terminology for female pelvic floor dysfunction, Neurourology and Urodynamics: Official Journal of the International Continence Society 29(1) (2010) 4-20.

[3] M.M. Van Dijk, H. Wijkstra, F.M. Debruyne, J.J. De La Rosette, M.C. Michel, The role of nocturia in the quality of life of men with lower urinary tract symptoms, BJU international 105(8) (2010) 1141-1146.

[4] A.P. Cameron, D.E. Chung, E.J. Dielubanza, E. Enemchukwu, D.A. Ginsberg, B.T. Helfand, B.J. Linder, W.S. Reynolds, E.S. Rovner, L. Souter, The AUA/SUFU guideline on the diagnosis and treatment of idiopathic overactive bladder, The Journal of urology 212(1) (2024) 11-20.

[5] G. Eginyan, X. Zhou, A.M.M. Williams, T. Lam, Effects of motor stimulation of the tibial nerve on corticospinal excitability of abductor hallucis and pelvic floor muscles, Frontiers in Rehabilitation Sciences 3 (2023) 1089223.

[6] J. Booth, L. Connelly, S. Dickson, F. Duncan, M. Lawrence, The effectiveness of transcutaneous tibial nerve stimulation (TTNS) for adults with overactive bladder syndrome: a systematic review, Neurourology and urodynamics 37(2) (2018) 528-541.

[7] H.A. de la Barra Ortiz, C. Cofré, C. López, I. Montecinos, Efficacy of diadynamic currents in the treatment of musculoskeletal pain: a systematic review, Physiotherapy Quarterly 31(3) (2023) 1-19.

[8] A. Demidaś, M. Zarzycki, Touch and pain sensations in diadynamic current (DD) and transcutaneous electrical nerve stimulation (TENS): a randomized study, Biomed Research International 2019(1) (2019) 9073073.

[9] S. Sayilir, M.T. Yildizgoren, The medium-term effects of diadynamic currents in chronic low back pain; TENS versus diadynamic currents: A randomised, follow-up study, Complementary therapies in clinical practice 29 (2017) 16-19.

[10] A. Uysal, M. Güntel, Comparison of diadynamic current, interferential current and transcutaneous electrical nerve stimulation therapies in patients with chronic low back pain, Deneysel ve Klinik Tıp Dergisi 39(4) (2022) 1078-1082.

[11] E.E.M. Abdelatief, B.A.N.A. Almaboud, Effect of premodulated interferential current versus diadynamic current on the management of lateral elbow tendinopathy, Biomedical Human Kinetics 15(1) (2023) 17-26.

[12] S. Kane, Sample Size Calculator. <https://clincalc.com/Stats/SampleSize.aspx>, 2025 (accessed 28-11-2025 2025).

[13] R.H. Jacomo, A.T. Alves, A. Lucio, P.A. Garcia, D.C.R. Lorena, J.B. de Sousa, Transcutaneous tibial nerve stimulation versus parasacral stimulation in the treatment of overactive bladder in elderly people: a triple-blinded randomized controlled trial, Clinics 75 (2020) e1477.

[14] D.-E. Taha, M. Elbaset, A. Hashem, H. Nabeeh, A. Ibrahim, M.N. Pardoe, A.S. Elhefnawy, Validation of the Arabic linguistic version of the International Consultation on Incontinence Questionnaire-overactive bladder (ICIQ-OAB), African Journal of Urology 27(1) (2021) 51.

[15] M.I. Maulana, Y.M. Sari, Factors Associated with Stress Urinary Incontinence During Pregnancy at Pasar Kuok Community Health Center, Andalas Obstetrics And Gynecology Journal 9(2) (2025) 224-230.

[16] S. Monteiro, C. Riccetto, A.K. Rocha, K. Santos, I. Campos, T. Pereira, S. Botelho, The Brazilian Portuguese version of the ICIQ-OABqol: cross-cultural adaptation and reliability, International Urogynecology Journal 31(12) (2020) 2507-2514.

[17] J. Tornic, M.D. Liechti, S.A. Stalder, V. Birkhäuser, S. van der Lely, L. Leitner, U. Mehnert, T.M. Kessler, Transcutaneous tibial nerve stimulation for treating neurogenic lower urinary tract dysfunction: a pilot study for an international multicenter randomized controlled trial, European urology focus 6(5) (2020) 909-915.

[18] D.A. Vaca-Benavides, W. Ju, C. Gonzalez, P. Aitken, A.K. Appukuttan Nair Syamala Amma, S. Mitra, S.D. Shenkin, The importance of electrical parameters on transcutaneous tibial nerve stimulation for overactive bladder syndrome: a systematic review and meta-analysis, Age and ageing 54(7) (2025) afaf203.

[19] Y. Sheng, J.S. Carpenter, J.A. Ashton-Miller, J.M. Miller, Mechanisms of pelvic floor muscle training for managing urinary incontinence in women: a scoping review, BMC women's health 22(1) (2022) 161.

[20] L.F.M. Pires, M.A.R. da Costa, E.U. Vitória, M.G. de Souza, L.R.L. Xavier, R.F. de Oliveira, Applicability of kegel exercises in different urinary incontinences in women: Systematic review, Manual Therapy, Posturology & Rehabilitation Journal 22 (2024).

[21] C.M.S. Katz, C.P. Barbosa, Effects of hypopressive exercises on pelvic floor and abdominal muscles in adult women: A systematic review of randomized clinical trials, Journal of Bodywork and Movement Therapies 37 (2024) 38-45.

[22] A. Pautz, M.C. Michel, Sex and gender differences in the pharmacology of the overactive urinary bladder, in: Sex and Gender Effects in Pharmacology, Springer2023, pp. 57-74.
